# Factors affecting the use of maternal health services in Madhya Pradesh state of India: a multilevel analysis

**DOI:** 10.1186/1475-9276-10-59

**Published:** 2011-12-05

**Authors:** Tej Ram Jat, Nawi Ng, Miguel San Sebastian

**Affiliations:** 1 United Nations Population Fund, Bhopal, Madhya Pradesh-462013, India; 2 Department of Public Health and Clinical Medicine, Epidemiology and Global Health, Umeå University, SE-90187, Sweden

**Keywords:** Maternal health, antenatal care, skilled attendance at delivery, post natal care, multilevel analysis

## Abstract

**Background:**

Improving maternal health is one of the eight Millennium Development Goals. It is widely accepted that the use of maternal health services helps in reducing maternal morbidity and mortality. The utilization of maternal health services is a complex phenomenon and it is influenced by several factors. Therefore, the factors at different levels affecting the use of these services need to be clearly understood. The objective of this study was to estimate the effects of individual, community and district level characteristics on the utilisation of maternal health services with special reference to antenatal care (ANC), skilled attendance at delivery and postnatal care (PNC).

**Methods:**

This study was designed as a cross sectional study. Data from 15,782 ever married women aged 15-49 years residing in Madhya Pradesh state of India who participated in the District Level Household and Facility Survey (DLHS-3) 2007-08 were used for this study. Multilevel logistic regression analysis was performed accounting for individual, community and district level factors associated with the use of maternal health care services. Type of residence at community level and ratio of primary health center to population and percent of tribal population in the district were included as district level variables in this study.

**Results:**

The results of this study showed that 61.7% of the respondents used ANC at least once during their most recent pregnancy whereas only 37.4% women received PNC within two weeks of delivery. In the last delivery, 49.8% mothers were assisted by skilled personnel. There was considerable amount of variation in the use of maternal health services at community and district levels. About 40% and 14% of the total variance in the use of ANC, 29% and 8% of the total variance in the use of skilled attendance at delivery and 28% and 8.5% of the total variance in the use of PNC was attributable to differences across communities and districts, respectively. When controlled for individual, community and district level factors, the variances in the use of skilled attendance at delivery attributed to the differences across communities and districts were reduced to 15% and 4.3% respectively. There were only marginal reductions observed in the variance at community and district level for ANC and PNC use. The household socio-economic status and mother's education were the most important factors associated with the use of ANC and skilled attendance at delivery. The community level variable was only significant for ANC and skilled attendance at delivery but not for PNC. None of the district level variables used in this study were found to be influential factors for the use of maternal health services.

**Conclusions:**

We found sufficient amount of variations at community and district of residence on each of the three indicators of the use of maternal health services. For increasing the utilisation of these services in the state, in addition to individual-level, there is a strong need to identify and focus on community and district-level interventions.

## Background

Improving maternal health is one of the eight Millennium Development Goals [1]. According to estimates developed by the WHO, UNICEF, UNFPA and the World Bank, there were estimated 358,000 maternal deaths globally during 2008. Of the total estimated maternal deaths, developing countries accounted for 99% maternal deaths and with 63,000 cases in the year 2008, India had the largest number of maternal deaths in the world [2,3].

In the year 2009, maternal mortality ratio (MMR) in India was 212 maternal deaths on every 100,000 live births. Madhya Pradesh state of India with 269 maternal deaths on every 100,000 live births was among the states with highest MMR in the country [4]. Some of the key reasons behind the high MMR in the state are low literacy levels among population, difficult geographic terrain in some parts of the state, inadequate availability and lower levels of utilization of emergency obstetric care services along with lower levels of utilization of antenatal care, safe delivery and post natal care services [5]. Inadequate availability of health infrastructure and resources along with huge economic inequity, gender disparities, societal norms and attitudes of community and service providers might be related to low levels of utilization of health care services in the state. The literacy rates in the state in 2011 were 80.5% for males and 60% for females respectively. The state government has set an ambitious target of reducing MMR to 220 per 100,000 live births by the year 2012. In order to achieve this target, the state government is focusing on increasing the use of maternal health care services including institutional deliveries, skilled attendance at delivery and strengthening emergency obstetric care services [5].

It is widely accepted that the use of maternal health services helps in reducing maternal morbidity and mortality. However, the utilisation of maternal health services is a complex phenomenon influenced by many factors. Various studies conducted worldwide [6-10] and in India [11,12] have recognized socio-economic factors and service delivery environment as important determinants for the use of maternal health services. A study on influence of community-level characteristics on the use of maternal and reproductive health services conducted in Uttar Pradesh state of India reported strong community-level influences on service use. This study further highlighted a need for looking beyond individual factors when examining health-care seeking behavior [12]. Positive relationship between level of maternal education and use of maternal health care services has been indicated by previous studies in India [13-15]. A study on the use of antenatal care services in Madhya Pradesh recorded significant association between the use of antenatal care and factors such as women's education, household's standard of living, cast and religion [14]. There is a need of clear understanding of the socio-cultural and service delivery related factors at different levels affecting the use of maternal health services. However, very little research in this regard is available in the context of Madhya Pradesh state of India.

The objective of this study was to estimate the effects of individual, community and district level characteristics on the utilisation of maternal health services with special reference to antenatal care, skilled attendance at delivery and postnatal care.

## Methods

### Study site

Madhya Pradesh is the second largest state in India with an area of 308,000 square kilometers. The state accounts for 9.4% of India's geographical area. According to the provisional figures of 2011 census, population of the state is 72,597,565. The decadal growth rate of population in Madhya Pradesh during 2001-2011 was 20.3% which was higher than that of India (17.64%). Population density of the state is 236 persons per square kilometer as against 382 for the country [16]. The population of the state is primarily rural with only 26.7% residing in urban areas. The state has 20.3% tribal and 16% scheduled cast population [17]. The state is one of the poorest six states of India and around 38% population of the state was living below poverty line in 2004-2005 [18].

The state comprises of 55,393 villages, 313 development blocks and 50 administrative districts. There are huge geographical, social, economic and cultural variations between and within districts [19]. The responsibilities of health care services lie with the department of public health and family welfare of the state government. In 2008, there were 270 community health centres, 1149 primary health centres and 8,834 health sub centres in the state to provide preventive and curative health care services in rural areas [20]. The state also has a huge network of private sector health care facilities which are mainly concentrated in urban areas.

### Study design and sampling technique

This study was designed as a cross sectional study. The data was derived from the District Level Household and Facility Survey 2007-08 (DLHS-3), a nationwide household survey conducted to obtain reproductive and child health outcome indicators. This survey employed a multi-stage stratified systematic sampling design. In each district of the state, 50 primary sampling units (PSUs) were selected which were villages in rural areas and census enumeration blocks (CEBs) in urban areas. In rural areas villages were selected by probability proportional to size (PPS) systematic sampling and in the second stage households were selected by systematic sampling. For urban areas first wards were selected by PPS systematic sampling, in the second stage CEBs by PPS sampling and households in the third stage by systematic sampling. All ever married women aged 15-49 years from sampled households were the respondents. The Madhya Pradesh sample of the DLHS-3 data had a sample size of 46,634 ever-married women between the ages of 15 and 49 years from 51,419 households in 2,246 primary sampling units selected from all districts of the state [21].

The word community is used in this study to describe clustering within the same geographical living environment. Communities were based on sharing a common primary sampling unit (PSU) within DLHS 3 data. PSUs were census villages in rural areas and census enumeration blocks in urban areas based on 2001 census. In order to make a proper rural PSU, selected villages with less than 50 households were linked with another contiguous village and selection probability was adjusted accordingly. Selected villages with more than 300 households were further divided into two or more parts and one or more parts were selected. Details of sampling techniques and sampling weights are published elsewhere [21]. The data was collected between December 2007 and December 2008.

For the analysis reported in this paper, a total of 15,782 ever-married women aged 15-49 years residing in Madhya Pradesh state who delivered a child during the three years preceding the survey were selected. Thus, 15,782 women from 2,246 primary sampling units in 45 districts were included in this study. In addition to using the individual data from DLHS-3, district level census data published by the Registrar General of India were also accessed [17] and data on rural health infrastructure in 2008 published by the Ministry of Health and Family Welfare, Government of India (MOH&FW, GOI) were also included in the analysis [20].

### Variables

#### Outcome variables

Three indicators of the use of maternal health services were used in this analysis: use of any antenatal care during pregnancy, skilled attendance at delivery (this included institutional deliveries and deliveries at home conducted by trained health personnel) and receiving any postnatal care (checkup by health professional within two weeks of delivery). As part of maternal health care services, ANC provided by a doctor, auxiliary nurse midwife or other health professional comprises of physical checks, checking growth of foetus and giving tetanus toxoide injection at periodic intervals during the pregnancy. These services are provided free of cost in all government health facilities in the state. In this study ANC was defined as receiving any one antenatal checkup during the most recent pregnancy during the last 3 years preceding the survey. The factors associated with each of these indicators were assessed separately and with reference to the most recent delivery.

#### Explanatory variables

The role of individual factors such as mother's age at last birth (15-24 years, 25-34 years and 35 years or older), mother's level of education (illiterate, primary-5 years of schooling, middle-8 years of schooling and higher secondary-12 years of schooling and above), mother's occupation, birth order (1, 2 and 3 or more), mother's cast (scheduled tribes, scheduled cast and others), mother's religion (Hindu, Muslim and others), socio-economic status, poverty index and husband's level of education (illiterate, primary, middle and higher secondary and above) were examined. Mother's occupation was categorized into three groups: unemployed, farmers/agriculture workers/laborers and professional/service/production worker.

Socio-economic status was based on wealth index divided into quintiles, which was computed based on the data from DLHS-3 survey by combining household amenities, assets and durables; households were categorized from the poorest to the richest groups corresponding from the lowest to the highest quintiles. Poverty index was measured by the possession of below poverty line (BPL) cards. This is a proxy for below or above poverty line households as per the National Planning Commission poverty threshold according to the 61st round of the National Sample Survey (NSS) and the criterion used for this threshold was monthly per capita consumption expenditure below Indian rupees 356.35 for rural areas and Indian rupees 538.60 for urban areas [18].

At the community level, the role of place of residence was assessed. Place of residence was defined as urban or rural. Two district level variables, percentage of tribal population and the ratio of primary health centre (PHC) to population in the district of residence, were included. Huge inequities in terms of availability of health facilities and human resources exist between tribal areas of the state in comparison with other areas with non-tribal population. The ratio of PHC to population was used as a proxy for the availability of health services in the district. These determinants of the use of maternal health services were selected based on information from existing literature [8,11-15]. While assessing the factors associated with skilled attendance at delivery, the use of antenatal care was also used as one of the variables. Use of antenatal care and skilled attendance at delivery were also included along with other variables for assessment of the correlates of the use of postnatal care.

### Statistical analysis

First, we calculated descriptive statistics. We presented the proportion of women who used each of the three maternal health services for each category in the explanatory variables. We also calculated the univariate odds ratio (OR) with 95% confidence interval (95% CI). To take into account the hierarchical structure of the sample, where individuals are nested within communities (PSUs) and communities are nested within districts, a logistic multilevel modeling was applied in this study [22-25]. Thus, a multilevel model with three levels was fitted to assess the influences of measured individual, community and district factors (fixed effects) on the use of maternal health services. In addition, we also estimated the community and district level random effects using the *xtmelogit *command in Stata 11 (Stata Corp. Inc., TX, USA). For each of the three outcome variables (use of antenatal care, skilled attendance at delivery and postnatal care), six models were estimated. In model 1 (empty model) no explanatory variable was included. This model represented the total variance in the use of three maternal health services between the communities and the districts. In Model 2, only individual-level factors were included. Model 3 and 4 were about the effects of only community level and district level factors respectively. In Model 5, community and district level factors were included together. Model 6 expanded on the previous model by adding the individual-level variables. In order to simplify the presentation of the findings, only model 1 and 6 are presented in the results section. The results of fixed effects (measures of association) are shown as odds ratios (ORs) with 95% confidence intervals (CIs). The results of random effects (measures of variation) are presented as variance partition coefficient (VPC) ^1^.

As this study used several explanatory variables that might be correlated to each other (such as mother's education, father's education and household wealth index), the multicollinearity assessment was conducted using the means of variance inflation factors (VIFs) as a post-estimation procedure following the regression analysis. The small VIF of 2.4 indicated the absence of any significant collinearity between explanatory variables in the regression model. The interaction between birth order and mother's age at last birth was also checked, and as no significant interaction term was identified, it was excluded from the regression models.

### Ethics

The study presented in this paper is based on analysis of existing survey data with all identifier information removed. The DLHS-3 survey was approved by the Ministry of Health & Family Welfare, Government of India and the International Institute of Population Sciences (IIPS) was nodal agency for conducting the survey. Informed consent was obtained from all study participants before participation and all information was collected confidentially. The raw data of DLHS-3 was used for this study with permission from the IIPS.

## Results

The results of this study showed that 61.7% of the respondents used antenatal services at least once during their most recent pregnancy. The percentage of mothers whose last delivery was assisted by skilled personnel was 49.8 whereas only 37.4% women received postnatal care within two weeks after their most recent delivery (Table 1).

**Table 1:** Variations and univariate odds ratio (with 95% confidence interval) indicators of the use of maternal health care services in Madhya Pradesh State of India

	N	Received any ANC		Skilled attendance at delivery		Received PNC within 2 weeks	
		**%**	**OR (95% CI)**	**%**	**OR (95% CI)**	**%**	**OR (95% CI)**

**All respondents**	**15782**	**61.7**		**49.8**		**37.4**	

***Individual variables***							

**Mother's age at last birth**							

= > 35	801	45.1	1	34.1	1	25.5	1

25-34	6223	60.0	1.83 (1.58-2.12)	47.2	1.73 (1.48-2.02)	36.2	1.66 (1.41-1.96)

15-24	8758	64.3	2.20 (1.90-2.54)	53.1	2.19 (1.88-2.55)	39.4	1.90 (1.61-2.24)

**Mother's level of education**							

Illiterate	8352	48.8	1	37.3	1	26.3	1

Primary	2775	67.2	2.15 (1.96-2.35)	51.3	1.77 (1.63-1.93)	37.8	1.70 (1.55-1.86)

Middle	2444	75.6	3.25 (2.93-3.59)	61.7	2.71 (2.47-2.97)	47.7	2.56 (2.33-2.81)

Higher Secondary & above	2211	87.9	7.64 (6.67-8.74)	82.0	7.67 (6.82-8.63)	67.7	5.89 (5.32-6.52)

**Mother's occupation**							

Unemployed	7681	68.7	1	61.6	1	45.7	1

Farmers/Agricultural Workers/Labourer	7313	53.0	0.52 (0.48-0.55)	36.7	0.36 (0.34-0.39)	27.7	0.46 (0.43-0.49)

Professional/Service/Production Worker	788	73.1	1.24 (1.05-1.46)	56.7	0.82 (0.70-0.95)	46.7	1.04 (0.90-1.21)

**Birth order**							

= > 3	5702	53.2	1	37.8	1	28.1	1

2	3853	67.8	1.86 (1.7-2.02)	52.2	1.80 (1.65-1.95)	40.9	1.77 (1.62-1.93)

1	6227	65.6	1.68 (1.56-1.81)	59.3	2.39 (2.22-2.58)	43.8	1.99 (1.85-2.15)

**Cast**							

Scheduled Tribes	4333	46.3	1	30.8	1	26.1	1

Scheduled Casts	2619	62.6	1.94 (1.76-2.14)	51.3	2.37 (2.14-2.62)	35.3	1.54 (1.39-1.71)

Others	8830	68.9	2.58 (2.39-2.78)	58.7	3.20 (2.96-3.46)	43.6	2.18 (2.02-2.37)

**Religion**							

Hindu	14954	60.7	1	48.7	1	36.6	1

Muslim	727	80.0	2.60 (2.16-3.13)	71.2	2.61 (2.22-3.08)	51.6	1.84 (1.59-2.14)

Others	101	71.3	1.61 (1.04-2.48)	63.4	1.82 (1.21-2.74)	56.4	2.24 (1.51-3.33)

**Household socio-economic status**							

Poorest	4548	42.7	1	32.0	1	23.7	1

Poor	4422	55.2	1.66 (1.52-1.80)	41.6	1.51 (1.39-1.65)	29.8	1.36 (1.24-1.50)

Medium	2787	68.3	2.90 (2.62-3.20)	54.1	2.51 (2.28-2.76)	39.8	2.13 (1.92-2.35)

Rich	2153	79.8	5.30 (4.69-5.97)	65.9	4.11 (3.69-4.59)	48.8	3.06 (2.74-3.41)

Richest	1872	92.0	15.5 (13.0-18.5)	87.6	15.0 (12.9-17.5)	72.0	8.28 (7.33-9.36)

**Poverty index**							

Possess BPL Card	6705	58.0	1	44.1	1	34.4	1

No BPL Card	9077	64.4	1.31 (1.22-1.39)	54.0	1.49 (1.4-1.59)	39.7	1.26 (1.18-1.34)

**Education level of husband**							

Illiterate	4661	45.4	1	34.0	1	24.5	1

Primary	2681	58.0	1.66 (1.51-1.83)	42.8	1.46 (1.32-1.60)	32.0	1.45 (1.30-1.61)

Middle	3271	66.1	2.34 (2.14-2.57)	52.5	2.15 (1.96-2.35)	37.8	1.87 (1.70-2.07)

Higher Secondary & above	5169	75.4	3.70 (3.40-4.03)	66.0	3.77 (3.47-4.10)	51.6	3.29 (3.01-3.58)

							

***Community level variables***							

**Type of residence**							

Rural	12653	56.5	1	43.1	1	32.3	1

Urban	3129	82.3	3.59 (3.25-3.96)	76.8	4.37 (3.99-4.78)	58.3	2.94 (2.72-3.19)

							

***District level variables ***							

**% tribal population in the district**							

> 50	1819	51.7	1	34.7	1	32.2	1

26-50	3395	62.6	1.56 (1.39-1.75)	44.1	1.48 (1.32-1.67)	35.7	1.17 (1.04-1.32)

0-25	10568	63.1	1.60 (1.45-1.77)	54.2	2.23 (2.01-2.47)	38.9	1.34 (1.21-1.49)

**Ratio of PHC to district population**							

> 30000	11963	62.0	1	52.5	1	38.4	1

Up to 30000	3819	60.5	0.94 (0.87-1.01)	41.5	0.64 (0.60-0.69)	34.4	0.84 (0.78-0.91)

**Source: **District Level Household and Facility Survey 2007-08 (DLHS-3), Data on the percent of tribal population came from Census of India 2001 and ratio of PHC to population in the district of residence was calculated from the data in the Bulletin on Rural Health Care System and Infrastructure in India, 2008

### Univariate logistic regression

The cross tabulation (Table 1) indicated that women delivering at younger age were more likely to use antenatal care, receive skilled attendance at delivery and use postnatal care. The findings further showed that the mothers used more antenatal care during the second order birth in comparison with the first order birth but the use of antenatal care decreased with birth order more than two. The levels of skilled attendance at delivery and postnatal care decreased steadily with increased birth order. The three indicators of the use of maternal health services increased sharply with increased levels of education of mother. This analysis also indicated that women from families living below poverty line were less likely to use maternal health services in comparison with women living in families above poverty line. Women from schedule tribes population and Hindu religion were least likely to be users of the three dimensions of maternal health services. The women who were farmers, agricultural workers and labourers were less likely to use maternal health services in comparison with professional women and unemployed.

Women living in urban areas tended to use the maternal health care services more than those living in rural areas. Levels of the utilisation of maternal health services were low in the districts with high percentage of tribal population. The utilisation of safe delivery services was 11% higher in the districts having a ratio of PHC to population of more than 30,000. No substantial difference in the utilisation of ANC and PNC based on this variable was found. The univariate odds ratios showed a lack of association between the ratio of PHC to district population and the use of ANC, and mothers' occupation (professional or production worker) and the use of PNC. All other variables used in this study were significantly associated with the use of the three maternal health services. Household socio-economic status was the strongest factor associated with the use of the three maternal health service indicators. Mother's level of education was the second most influential factor for the use of maternal health services.

### Multilevel models

The first step in the multilevel model analysis was to consider if our data justified the decision to assess random effects at the community and district levels. The results of the random intercept only model are shown in Table 2 (empty model). There was a significant amount of variation in the use of three maternal health care indicators across the communities and the districts. Based on the variation partition coefficient (VPC) values, 40% and 14% of the total variance in the use of antenatal care, 29% and 8% variance in the use of skilled attendance at delivery and 28% and 8.5% variance in the use of postnatal care were attributable to the differences across communities and districts respectively. The largest variation at community and district level was observed for ANC, but smaller for the other two indicators. Table 3 shows the results of the final model when individual, community and district level variables were included together. Both fixed and random effects are included. When controlled for individual, community and district level factors, the variances in the use of skilled attendance at delivery attributed to the differences across communities and districts were reduced to 15% and 4.3% respectively. There were only marginal reductions observed in the variance at community and district level for ANC and PNC use.

**Table 2:** Parameter coefficients for the multilevel model for various indicators of the use of maternal health services - Empty model, without covariates

	Antenatal care	Skilled attendance at delivery	Postnatal care
***Random effects ***			

Community (PSU) random variance (SE^a^)	1.436 (0.089)	0.956 (0.063)	0.875 (0.061)

Community (PSU) VPC^b ^(%)	40.0	28.6	27.8

District random variance (SE)	0.762 (0.173)	0.364 (0.085)	0.389 (0.090)

District VPC (%)	13.9	7.9	8.5

^a ^SE: Standard error

^b ^VPC: variance partition coefficient

**Table 3:** Results of the multilevel analysis of the variables related to the use of maternal health services in Madhya Pradesh (15,782 Women from 2,246 Primary Sampling Units in 45 Districts)

Characteristics	Odds Ratios (Confidence Interval)		
	
	Antenatal Care	Skilled attendance at delivery	Postnatal care
***Fixed Effects ***			

***Individual variables***			

**Mother's age at last birth**			

= > 35	ref	ref	ref

25-34	1.27 (1.05-1.53)	1.13 (0.93-1.36)	0.95 (0.73-1.23)

15-24	1.28 (1.05-1.55)	1.22 (1.01-1.48)	0.86 (0.65-1.11)

**Mother's level of education**			

Illiterate	ref	ref	ref

Primary	1.37 (1.21-1.54)	1.20 (1.07-1.33)	1.00 (0.86-1.15)

Middle	1.69 (1.47-1.94)	1.44 (1.26-1.63)	1.17 (0.99-1.37)

Higher Secondary & above	2.57 (2.11-3.12)	2.35 (1.98-2.79)	1.39 (1.14-1.70)

**Mother's occupation**			

Unemployed	ref	ref	ref

Farmers/Agricultural Workers/Labourer	0.89 (0.80-0.98)	0.72 (0.66-0.79)	0.92 (0.81-1.04)

Professional/Service/Production Worker	1.37 (1.10-1.70)	0.83 (0.68-1.00)	0.97 (0.77-1.23)

**Birth order**			

= > 3	ref	ref	ref

2	1.37 (1.21-1.53)	1.19 (1.06-1.32)	1.11 (0.95-1.27)

1	1.69 (1.51-1.90)	1.87 (1.68-2.07)	1.27 (1.11-1.46)

**Cast**			

Scheduled Tribes	ref	ref	ref

Scheduled Casts	1.64 (1.41-1.91)	1.52 (1.32-1.74)	0.85 (0.70-1.03)

Others	1.53 (1.33-1.73)	1.41 (1.24-1.59)	0.92 (0.77-1.08)

**Religion**			

Hindu	ref	ref	ref

Muslim	1.52 (1.16-1.97)	1.26 (1.01-1.56)	0.81 (0.63-1.03)

Others	0.79 (0.41-1.48)	0.96 (0.53-1.73)	1.46 (0.75-2.83)

**Household socio-economic status**			

Poorest	ref	ref	ref

Poor	1.16 (1.04-1.29)	1.07 (0.96-1.18)	0.99 (0.85-1.14)

Medium	1.54 (1.34-1.76)	1.15 (1.01-1.30)	1.13 (0.95-1.35)

Rich	2.16 (1.80-2.57)	1.21 (1.03-1.41)	1.03 (0.84-1.26)

Richest	4.53 (3.47-5.91)	2.32 (1.86-2.90)	1.50 (1.16-1.93)

**Poverty index**			

Possess BPL Card	ref	ref	ref

No BPL Card	0.90 (0.82-0.98)	0.98 (0.90-1.06)	0.88 (0.79-0.98)

**Education level of husband**			

Illiterate	ref	ref	ref

Primary	1.21 (1.06-1.36)	1.06 (0.94-1.19)	1.03 (0.87-1.21)

Middle	1.38 (1.21-1.56)	1.15 (1.02-1.29)	0.98 (0.83-1.15)

Higher Secondary & above	1.45 (1.27-1.66)	1.22 (1.08-1.38)	1.14 (0.96-1.35)

**Received any ANC**			

Not received	-	ref	ref

Received	-	3.52 (3.21-3.84)	2.62 (2.31-2.95)

**Skilled assistance at delivery**			

Delivery by unskilled personnel	-	-	ref

Delivery by skilled personnel	-	-	58.32 (50.20-67.74)

***Community level variables***			

**Type of residence**			

Rural	ref	ref	ref

Urban	1.64 (1.35-1.98)	1.88 (1.62-2.18)	0.94 (0.78-1.11)

***District level variables ***			

**Percent of tribal population in the district of residence**			

> 50	ref	ref	ref

26-50	1.23 (0.48-3.10)	0.95 (0.57-1.55)	0.60 (0.26-1.35)

0-25	0.89 (0.35-2.21)	1.07 (0.65-1.75)	0.52 (0.23-1.16)

**Ratio of PHC to population in the district**			

> 30000	ref	ref	ref

Up to 30000	1.04 (0.53-2.02)	0.75 (0.52-1.07)	1.03 (0.57-1.84)

***Random effects***			

Community (PSU) random variance (SE^a^)	0.977 (0.071)	0.410 (0.042)	0.541 (0.065)

Community (PSU) VPC^b ^(%)	32.90	14.92	23.68

District random variance (SE)	0.636 (0.145)	0.167 (0.041)	0.480 (0.111)

District VPC (%)	12.97	4.32	11.13

^a ^SE: Standard error

^b ^VPC: variance partition coefficient

#### Antenatal care

After adjusting for other variables in the multilevel model, both variables used at district level became insignificant for the use of ANC. Non-possession of BPL card was positively associated with the use of ANC in the univariate model whereas the results of multilevel analysis showed a negative association of this variable with the use of ANC. Results of the univariate model and the multilevel analysis showed that household socio-economic status and mother's education were the strongest individual level factors related to the use of antenatal services. The results of the multilevel model showed that women from richest quintile had 4.53 times more likelihood of receiving ANC during pregnancy in comparison with women from the poorest quintile of the society. The odds of reporting use of ANC by women with higher secondary and above education were 2.57 times higher than that of illiterate women. Other predictors of the use of antenatal care were mother's age at last birth, husband's education, mother's occupation, birth order, mother's cast and poverty index. Women who were younger than 35 years at the time of last birth were 1.28 times more likely to receive ANC than women in the age group of 35 years or more. Muslim and non-scheduled tribes women were more likely to receive ANC. At community level, urban residence had considerable positive association with the use of ANC but no relationship was found with the variables used at the district level. This indicates that urban residence has an independent effect on antenatal care, over and above the well-known effects of individual factors.

The data further showed that the VPC was still appreciably large; indicating that even after controlling for individual, community and district factors, there was a considerable clustering of antenatal care utilization at the community and district level. However, the community and district level correlates used in this study did not explain such variation.

#### Skilled attendance at delivery

The results of our study indicated that mother's level of education, use of ANC and household socio-economic status were the strongest factors associated with of the use of skilled attendance at delivery. The results of full model showed that women with higher secondary and above education were 2.35 times more likely to receive skilled attendance at delivery in comparison with illiterate women. Women having received at least one ANC during pregnancy had 3.52 times higher odds of receiving skilled attendance at delivery than women who did not receive any ANC. Women from the richest quintile of the society were also more likely to receive skilled attendance at delivery than women from the poorest quintile. Older women, women having more than 3 children, belonging to schedule tribes, to be Hindu and to be married to a low educated husband were associated with lower use of skilled attendance at delivery.

At community level, urban residence was found to be positively associated with receiving skilled attendance at birth. The district level variables, percentage of tribal population in the district of residence and ratio of PHC to population, did not show much influence on the use of skilled attendance at delivery. After adjusted for other variables in the multilevel model, both district level variables along with the variables; poverty index, primary level education of husband, other religions, mother's occupation being professional and mother's age at last birth between 25 to 34 became insignificant.

#### Postnatal care

The overall use of postnatal care was found to be very low. In the multilevel analysis, only some of the individual level variables such as mother's level of education, birth order one, mother being from the richest quintile and non possession of BPL card remained to be significant variables. The results of multilevel analysis showed that the stronger factor related to the use of PNC was skilled attendance at delivery (adjusted OR = 58.32; 95% CI 50.20-67.74). Further, the results showed that women from the richest households had higher likelihood of using postnatal care than women from the poorest households. The odds of reporting the use of postnatal care among women who were higher secondary and above educated were about 1.39 times higher than among those who were illiterate. Likewise, association was found between the use of PNC and birth order and use of ANC during pregnancy. Unlike the previous two maternal health indicators, postnatal care was not much influenced by the place of residence. Both district level variables could not show any significant association with the use of PNC in the multilevel analysis.

## Discussion

The association between individual, community and district level factors and the utilisation of maternal health services covering three aspects of maternal health care-use of antenatal care, skilled attendance at delivery and postnatal care were examined in this study. The use of maternal health services in Madhya Pradesh state remains low in comparison with many other Indian states. For example, the analysis in this study showed that 61.7% women received any ANC, 49.8% women received skilled attendance at delivery and 37.4% received any PNC within two weeks of delivery in Madhya Pradesh. The DLHS-3 showed that Kerala had almost universal coverage in the use of maternal health services, including ANC (99.8%) and the use of skilled attendance at delivery (99.4%). Similarly, high figures were found in the states of Tamilnadu and Andhra Pradesh [26].

The multilevel analysis has shown that individual-level, community-level and district-level factors are important factors associated with the use of maternal health care services in Madhya Pradesh state of India. The multilevel framework demonstrated significant community and district variations in the use of maternal health services. However, the district level variables used in this analysis were not found to be the influential factors for the use of maternal health services. Some of the variables found to be significant in univariate model became insignificant when adjusted for other variables in the multilevel analysis. A study on influence of community-level characteristics on the use of maternal and reproductive health services conducted in Uttar Pradesh state of India reported strong community-level influences on service use, although the type of community effect varied by service type. This study further highlighted that the role of some individual and household factors in determining a person's use of services was mediated by the characteristics of the community in which the individual lives. The results of this study demonstrated the need to look beyond individual factors when examining health-care seeking behavior [12].

The results of our study showed very strong positive influence of higher household socio-economic status on the use of all three indicators of maternal health services. Previous studies have also reported a positive association between socio-economic status and antenatal care, skilled attendance at delivery and postnatal care [13,15,27-30].

Findings regarding high influence of higher education levels of women on the use of maternal health services are consistent with other studies in India and other countries; the better educated women are, more aware about their health, know more about availability of maternal health care services and use this awareness and information in accessing the health care services [14,15,29,31,32]. Education of husband might be playing a similar role in supporting the women's access to the health services. In the rural areas of the state, maternal health services are delivered through government run CHCs, PHCs and Sub Health Centres. In urban areas, these services are rendered by medical colleges, district and civil hospitals and urban health posts. Maternal health services from private hospitals, nursing homes, health centers and private practitioners are also availed in rural and urban areas. Access to and availability of health care services is expected to be greater in the urban areas. The findings of our study regarding stronger influences of urban residence on the use of ANC services and skilled assistance at delivery are consistent with the results of previous studies [15,27,30]. No considerable differences in the use of skilled attendance at delivery were found between employed and unemployed women.

Religion and cast showed considerable influences on the use of ANC and safe delivery services whereas no noteworthy influence of these factors was found on post natal care in our study. A multi state study conducted in southern India demonstrated that caste had varied influence on the use of maternal health care services in different states; for example, this study reported that belonging to a lower caste was a stronger correlate of institutional delivery in Andhra Pradesh while in other states being a member of scheduled caste or tribe reduced the likelihood of using maternal health services [15]. Influence of religion and caste on the use of maternal health services needs to be further investigated. The findings of our study suggest that the use of ANC had a noteworthy effect on the use of skilled attendance at delivery and the use of both ANC and skilled attendance at delivery had considerable influence on the use of PNC. Another study conducted in rural India also recorded similar findings [33]. Our study found that the use of PNC was not much influenced by the place of residence. This may be because all women who deliver their babies in health facilities are given PNC before discharging them and providing or seeking PNC is negligible in the cases of home deliveries regardless of residence in urban or rural area.

This study showed no substantial influence of the ratio of PHC to population at district level on the use of maternal health services. This might be because the districts which have larger numbers of residents per PHC also have better presence of private health facilities. A study conducted in Madhya Pradesh in 2007 reported that out of 24,807 qualified doctors and 94,026 qualified paramedical staff mapped in the survey in the state, 18,757 (75.6%) and 67,793 (72.1%) were working in the private sector respectively [34]. Apart from these qualified medical and paramedical personnel, a huge network of unqualified practitioners also constitutes a huge part of private sector health care in the state. The role of private health care providers in influencing the use of maternal health services needs to be further studied. It is also surprising that despite having high shortfall of health facilities and human resources for health care in tribal areas of the state, no noteworthy influence of percent tribal population in the district of residence was found on the use of maternal health services. This issue needs to be further investigated. Further research is also required to identify the district level factors associated with the use of maternal health services as none of the variables used in this study at this level were found to be influential. The role of economic development, population-health personnel ratio, status of gender equity and women's empowerment at district level may be explored in this regard.

The findings of our study have implications on evidence based programming for maternal health care. These findings highlighted the need of adopting multilevel approaches along with addressing the factors affecting the use of maternal health services at individual, community and district levels. The amount of variation at community and district level found in our study indicates the need to contextualize efforts for increasing the use of maternal health services. Our study also revealed the existence of some unmeasured factors at community and district level influencing the utilisation of maternal health services. Hence, adopting district specific strategies along with identifying and addressing district level factors affecting the use of maternal health services would give better results.

## Limitations

The study had some limitations, which need to be considered when interpreting the results. The DLHS-3, the source of data for this study, was based on the self reported information of respondents and no validation of the provided information was done from other objective sources. Although there are concerns about self reported behaviour, it is reasonable to assume that biases are less likely in maternal health care related events in comparison with other sensitive issues such as sexual behaviour. Second, the data used for this analysis were from a cross sectional survey, therefore, we could only examine the association between explanatory variables and three indicators of the use of maternal health services and could not draw conclusions about causality. It might be possible that the relationships found are due to the influence of unmeasured individual, community and district level variables that are associated with both the dependent and independent variables in our estimated models. Third, some correlates of maternal health care utilisation are missing from our analysis such as distance of health facilities from the locality of residence, and this could have influenced the patterns of utilisation of maternal health services. Since data on this variable was not available in the survey, we used the ratio of PHC to population in the district as a proxy.

## Conclusions

In this study, we went beyond the individual factors and investigated the effects of community and district level factors on the utilisation of maternal health services. At community level, residence in urban area was consistently associated with increased likelihood of the utilisation of maternal health services, except for PNC. We found sufficient amount of variation at community of residence and district of residence on each of the three indicators of the utilisation of maternal health services.

Analysis of factors affecting the use of maternal health services revealed interesting findings, which have very important implications for evidence based programming for maternal health. The household socio-economic status and mother's education were the most important factors associated with the use of ANC and skilled attendance at delivery, therefore empowering women and promoting mother's education would yield greater results in increasing the use of maternal health services. The results of our study showed that, the use of ANC increases the odds of skilled attendance at delivery, which later increases the use of PNC. Hence it is very important to promote the use of ANC among pregnant women. The access of certain groups such as Hindus and scheduled tribes to maternal health services should be further emphasized.

In order to increase the utilisation of maternal health services in the state, in addition to individual-level, there is a strong need to focus on community and district-level interventions. Future research should investigate those factors that may account for the unexplained community and district variations in the use of maternal health services.

## Competing interests

The authors declare that they have no competing interests.

## Authors' contributions

TRJ and MSS conceptualized the study. TRJ acquired the raw data for analysis. TRJ, MSS and NN participated in the analysis of data. TRJ prepared the first draft of the manuscript. All three authors worked for revising various drafts of the manuscript. All authors read and approved the final manuscript.

## Endnotes

^1^In three-level logistic regression models, the VPC is calculated as: VPC_d _= $\text{VP}{\text{C}}_{\text{d}}\text{ = }\frac{\sigma_{\text{d}}^{2}}{\sigma_{\text{d}}^{2}+\sigma_{c}^{2}+3.29}$ and $\text{VP}{\text{C}}_{\text{c}}\text{ = }\frac{\sigma_{\text{d}}^{2}+\sigma_{c}^{2}}{\sigma_{\text{d}}^{2}+\sigma_{c}^{2}+3.29}$ where $\sigma_{c}^{2}$ represents community level variance and $\sigma_{\text{d}}^{2}$ represent the district level variance.
